# Psychological Distress in Patients with Autoimmune Arthritis during the COVID-19 Induced Lockdown in Italy

**DOI:** 10.3390/microorganisms8111818

**Published:** 2020-11-18

**Authors:** Andrea Picchianti Diamanti, Maria Sofia Cattaruzza, Roberta Di Rosa, Flavia Del Porto, Simonetta Salemi, Maria Laura Sorgi, Luis Severino Martin Martin, Alessandra Rai, Dalila Iacono, Giorgio Sesti, Guido Alessandri, Bruno Laganà

**Affiliations:** 1 Department of Clinical and Molecular Medicine, Sant’Andrea University Hospital, Sapienza University of Rome, 00185 Rome, Italy; roberta.dirosa@uniroma1.it (R.D.R.); flavia.delporto@uniroma1.it (F.D.P.); Simonetta.salemi@ospedalesantandrea.it (S.S.); marialaura.sorgi@uniroma1.it (M.L.S.); alessandra_rai@hotmail.it (A.R.); dalilaiacono@gmail.com (D.I.); giorgio.sesti@uniroma1.it (G.S.); bruno.lagana@uniroma1.it (B.L.); 2 Department of Public Health and Infectious Diseases, Sapienza University of Rome, 00185 Rome, Italy; mariasofia.cattaruzza@uniroma1.it; 3 Ospedale Regina Apostolorum, U.O. di Medicina Interna e Reumatologia, 00041 Albano Laziale, Italy; severino.martin@gmail.com; 4 Department of Psychology Sapienza, University of Rome, 00100 Rome, Italy; guido.alessandri@uniroma1.it

**Keywords:** COVID-19, chronic autoimmune arthritis, DASS-21, CPDI, psychological distress, depression, anxiety, disease activity

## Abstract

Lockdowns imposed by governments worldwide as a way to limit the spread of severe atypical respiratory syndrome-coronavirus-2 (SARS-CoV2) have had heavy psychological and economic consequences. Arthritis patients are a vulnerable population at an increased risk of peritraumatic stress. This could be due to several reasons, including the fear of shortage of medicine and difficulty receiving periodical medical checks. In the present case-control study, psychological distress in patients with autoimmune arthritis during the coronavirus disease 2019 (COVID-19) pandemic were investigated. An electronic survey was conducted to gather information on the perceived change in the emotional state, general health (GH), fatigue, joint pain, and disease activity during the lockdown, in 100 patients with autoimmune arthritis and 100 controls. Mental health status was measured using the Depression, Anxiety and Stress Scale (DASS-21). The COVID-19 Peritraumatic Distress Index (CPDI) was used to assess the frequency of peritraumatic stress disorders related to COVID-19. Patients reported a significant worsening of perceived GH (36% vs. 7%; *p* < 0.001), a significantly higher mean CPDI score (*p* < 0.001) than controls. Using multivariate analysis, arthritis patients had significantly higher CPDI scores (+3.67 points; *p* = 0.019), independent of depression, anxiety, and stress symptoms, comorbidities, and sociodemographic and lifestyle characteristics. Logistic regression analysis showed that the risk of reporting worsened GH was 9-fold higher in patients than controls (*p* < 0.001). Patients with autoimmune arthritis are at higher risk of psychological distress related to COVID-19 pandemic; thus targeted intervention should be designed to strengthen coping capacity in this vulnerable population.

## 1. Introduction

At the end of December 2019, after a cluster of cases of pneumonia of unknown origin were reported in the city of Wuhan, Hubei province, China, a new disease—coronavirus disease 2019 (COVID-19)—was identified [1].

Clinical manifestations of the disease are usually mild, and consist of fever, cough, and fatigue; however, 15–20% of patients can progress to severe or critic form that, not only require hospitalization, but can be complicated by Acute Respiratory Distress Syndrome (ARDS) with high mortality rate [2,3].

Since the outbreak, the virus has rapidly become a global emergency, due to how quickly it can spread, its sudden and unpredictable worsening of clinical course, and lack of effective therapy and prophylaxis. The World Health Organization (WHO), consequently, declared a pandemic on 11 March 2020 [4]. This new threat has led to worldwide fear over the virus, not only for the virulence and severity of the infectious agent, but for the characteristic of it, being an unknown invisible enemy, something that has never been encountered before. This aspect has been observed by recent studies questioning hastily published material. This was the case for the Angiotensin Converting Enzymes (ACE)2 receptor used by the virus to infect humans, and the possible interference by ACE2-inhibitors, which created worry in patients taking these drugs. Patients overcame these worries after reassuring studies emerged, although these were soon retracted due to the impossibility of accessing the documents [5,6]. Similarly, some possible treatments that were initially considered useful were deemed useless, or even dangerous, following rigorous and controlled studies [7,8]. Finally, there has been uncertainty about the duration of neutralizing protective antibodies, which casts doubt on the effectiveness of a vaccine [9]. The fear of COVID-19, at times, felt disproportionate compared to the real estimated risk of infection or mortality [10]. It has led to serious consequences across the globe, especially in patients with chronic diseases (e.g., autoimmune diseases, asthma, chronic obstructive pulmonary disease (COPD), cancer, psychiatric and cardiovascular diseases, etc.), who are strongly dependent on the need to have easy access to hospitals for periodical checks or, even more, for emergency situations.

Italy was the first country (outside of Asia) affected by the rapid spread of COVID-19, which led, at the end of March, to the highest number of deaths worldwide [11]. As a consequence, in an unprecedented attempt to delay the spread of the virus, a hard lockdown was imposed on the whole Italian territory on 10 March [12], independent of the epidemiological situation, which was markedly different at a national level, with the north particularly hit, and the center, south, and the islands relatively spared. Thus, in the inhabitants of the regions relatively spared by the virus, the hard lockdown was experienced even worse, as it may have been considered a measure disproportionate to the threat. Similar restrictive regimens were then adopted worldwide. Lockdown measures in Italy led to the closure of schools, universities, and all non-essential businesses; people were required to stay at home, and only leave the house for proven reasons of need (e.g., health or work). Protocol violations were liable to fines or penalties (up to 3 months in prison). These measures affected many aspects of peoples’ lives, regarding health, psychological, social, and economic consequences. A recent review showed that quarantined individuals are more likely to observe psychological distress, which seems to be determined, in particular, by quarantine duration and fear of infection [13]. Different studies, originally conducted by Chinese and Iranian groups, and then confirmed in Italian and Spanish populations, revealed a wide range of psychosocial distress at the individual and community levels, such as panic disorder, anxiety, and depression as a consequence of the lockdown [14,15,16,17,18,19]. From these studies, what emerged was that psychological distress may be predictable in the female gender, whereas other demographic and social characteristics were inconstantly found to be associated to psychological distress, such as age, education, and family composition. Finally, affective temperament and adult attachment style features were found to predict the extent of the mental health burden [20].

Autoimmune chronic arthritis, such as rheumatoid arthritis (RA) and spondyloarthritis (SpA), are chronic, inflammatory diseases associated with functional impairment and deterioration in health-related quality of life (HR-QoL) and life expectancy [21,22]. In Italy, RA affects between 0.41 and 0.48% of the population and the prevalence of SpA is similar, with ankylosing spondylitis at 0.37% and psoriatic arthritis at 0.42%, accounting for a total of about 700.000–800.000 Italians, confirming the importance of autoimmune arthritis as a public health issue [23,24]. It has already been demonstrated that both depression and anxiety are prevalent in these patients with respect to the general population, and a particular need for mental healthcare in this population has been proposed [25,26,27,28,29,30,31,32,33]. The management of patients affected by chronic diseases, such as arthritis, has become significantly more complex in this COVID-19 scenario, for a number of reasons. At first, the impossibility to carry out clinical activities during the lockdown, and the consequential logistical difficulties to reschedule all non-emergency visits, has led to a severe disadvantage for both patients and physicians. The impracticability of exerting a tight control strategy was potentially responsible for the worse disease outcome and a higher risk of flare-ups in arthritis patients. Difficulty in receiving appropriate and timely visits, and the fear of shortage of supplies of some immunosuppressive drugs (even used in patients affected by severe atypical respiratory syndrome-coronavirus-2 (SARS-CoV-2) to dampen the uncontrolled inflammatory immune response of the host [34,35,36]), such as hydroxychloroquine and tocilizumab, could have contributed toward triggering distress symptoms, and inducing a reduction in the adherence to, and compliance with, therapy. Finally, arthritis patients who are susceptible to severe infections, especially as a consequence of long-term immunosuppressive therapy, might feel fearful of being at a higher risk of SARS-CoV-2 infection (and severe infection consequences); thus increasing their anxiety, even though, as yet, data do not support this belief [37,38,39]. For all of the above considerations, autoimmune arthritis is a type of pathology that, like other chronic diseases, may particularly be affected by the negative consequences of the COVID-19-induced lockdown.

This is the first study, to the best of our knowledge, aimed at addressing the level of psychological distress in patients with autoimmune arthritis during the COVID-19 pandemic compared to the control population in Italy.

## 2. Materials and Methods

All subjects gave their written informed consent for inclusion before they participated in the study. The study was conducted in accordance with the Declaration of Helsinki, and the protocol was approved by local ethics committee (rif.7009_2020, May 2020).

### 2.1. Inclusion Criteria

Criteria included: being older than 18, living in Italy, experiencing the lockdown (imposed by the government) at home, and not being suspected (or confirmed) of having COVID-19.

For autoimmune arthritis patients (RA and SpA)—having a confirmed diagnosis according to the European League Against Rheumatism (EULAR)/American College of Rheumatology (ACR) [40] and the Assessment of Spondyloarthritis International Society(ASAS) 2010 criteria [41].

### 2.2. Procedures

From 27 April to 30 April 2020, an anonymous questionnaire, developed using Google Forms, and designed to assess psychological responses during the COVID-19 pandemic, was sent to autoimmune arthritis patients attending the S. Andrea University Hospital Immuno-Rheumatology outpatient clinic, and to a convenient sample of controls selected among relatives of people working in the same hospital, to ensure that the catchment area of cases and controls were similar.

For patients and controls, sociodemographic and lifestyle data (gender, age, height, weight, education, employment, living alone, smoking, alcohol drinking, and physical activity) were collected.

Inferences about changes from the beginning of the lockdown in perceived general health (GH), fatigue, emotional status, and (only for patients) joint pain and disease activity, five additional items were investigated. Each item had five responses on the Likert scale, 1 being “a really better change”, 2 “a better change”, 3 “no change”, 4 “a worse change” and 5 “a really worse change”. This format was adapted from the short-form survey-36 (SF-36) questionnaire [42].

Mental health status was measured using the Depression, Anxiety, and Stress Scale (DASS-21), a standardized and validated scale already used in chronic arthritis patients, as well as in previous research related to SARS and COVID-19 [43,44]. The total depression subscale score was divided into normal (0–9), mild depression (10–12), moderate depression (13–20), severe depression (21–27), and extremely severe depression (28–42). The total anxiety subscale score was divided into normal (0–6), mild anxiety (7–9), moderate anxiety (10–14), severe anxiety (15–19), and extremely severe anxiety (20–42). The total stress subscale score was divided into normal (0–10), mild stress (11–18), moderate stress (19–26), severe stress (27–34), and extremely severe stress (35–42).

To investigate the assumption that psychological distress was closely related to the COVID-19 scenario, a questionnaire specifically dedicated to address the peritraumatic psychological impact of COVID-19 was added. In the absence of universally accepted scales fit for purpose, the COVID-19 Peritraumatic Distress Index (CPDI), already adopted in Chinese and Iranian general populations [14,17], translating the English version into Italian, was adopted. Thereafter, the Italian version of the CPDI was validated by an Italian group [45]. CPDI is an index of 24 questions designed to assess the frequency of specific phobias and stress disorders related to COVID-19, including anxiety, depression, cognitive change, avoidance and compulsive behavior, physical symptoms, and loss of social functioning in the past week [17]. CPDI ranges from 0 to 100; a score between 28 and 51 indicates mild to moderate distress, a score ≥ 52 indicates severe distress.

The presence of comorbidity was also assessed, and patients were asked to report current therapy and difficulties in supply of rheumatologic drugs.

### 2.3. Statistical Analysis

In order to summarize data and evaluate differences, percentages and χ^2^ tests were used for categorical variables, while means, standard deviation (sd), and unpaired t-test were used for continuous variables. Multivariable analyses were used to evaluate factors associated with outcomes and to adjust for the effect of confounders. In particular, in multilinear regression, CPDI score was used as the dependent variable, while socio-demographic (age, gender, body mass index (BMI), education, employment), lifestyle (smoking, alcohol use, living alone), and clinical (being a case, having comorbidity, depression, anxiety, stress) independent variables were used to investigate their effects on CPDI.

The multivariate logistic regression was used to investigate factors independently associated with self-reported worsening of perceived GH. Since this variable had 5-response choices on the Likert scale ranging from 1 (really better now) to 5 (really worse now), it was dichotomized 1–3 and 4–5, and used as the dependent variable. The socio-demographic (age, gender, BMI, education, employment), lifestyle (smoking, alcohol use, living alone), and clinical (being a case, having comorbidity, depression, anxiety, stress) variables, plus the CPDI score, were used as independent variables in order to investigate their independent effects on the perceived GH.

*p*-values ≤ 0.05 were considered statistically significant.

## 3. Results

The survey was completed by 100 patients and 100 controls. Demographic, lifestyle, and clinical characteristics of arthritis patients and controls are reported in Table 1.

The analysis of demographic and lifestyle variables between patients and controls showed the following statistically significant differences: patients presented a higher prevalence of female gender (*p* = 0.005), were older (*p* = 0.003), had higher BMI score (*p* = 0.032), included more current smokers (*p* = 0.005), alcohol drinkers (*p* = 0.016), were affected by more comorbidities (*p* = 0.007). In particular, comorbidities were represented by blood hypertension and cardiovascular diseases, diabetes, and thyroid diseases, which were present in 23, 5, and 12 patients vs. 20, 2, and 6 controls, respectively, whereas autoimmune arthritis was present in all the patients and absent in controls.

The DASS-21 depression, anxiety, and stress score means for arthritis patients were all significantly higher than those for controls. Moreover, the percentages of patients with severe/extremely severe depression, anxiety and stress were higher than those of controls. In particular, 15% of patients vs. 4% of controls presented severe/extremely severe depressive symptoms, 16% of patients vs. 8% of controls reported severe/extremely severe anxiety symptoms, and 15% of patients vs. 5% of controls reported severe/extremely severe stress symptoms.

The mean CPDI score was significantly higher in patients who had an average of 6.3 points higher than controls (*p* < 0.001). The percentage of patients with a score indicating mild–moderate presence of COVID-19 peritraumatic distress was significantly higher than that of controls (38% vs. 22%; *p* = 0.022). A significantly higher percentage of patients reported a worsening in the perceived GH (36% vs. 7%; *p* < 0.001).

Ten percent of the patients (all receiving hydroxychloroquine) reported difficulty in drug supply.

After adjusting for all of the socio-demographic and lifestyle variables (age, gender, BMI, education, employment, smoking, alcohol use, living alone), the variables “suffering from depression”, “anxiety”, or “stress” significantly increased the CPDI score of 4.7, 8.0, 7.6 points, respectively. Being an arthritis patient increased the CPDI score of 3.8 points (*p* = 0.014), independently from the presence of depression, anxiety, and stress. All of the other variables did not seem to influence the CPDI score in a statistically significant way (Table 2).

Moreover, results from the logistic regression analysis (Table 3), after controlling for potential confounders, (socio-demographic and lifestyle variables, comorbidities, the presence of depression, anxiety, stress and COVID-19 peritraumatic distress), showed greater risk to report a worsening of the GH during the lockdown for arthritis patients, in comparison to controls. Indeed, arthritis patients were 9-fold more likely to report a worsening of their GH, in comparison to controls, after adjusting for sociodemographic and lifestyle variables, comorbidities, the presence of depression, anxiety, stress and COVID-19 peritraumatic distress. Independently from being an arthritis patient, the presence of a CPDI score, implying a mild, moderate, or severe distress, had a significantly 5.6 greater risk of reporting a worsening of the GH during the lockdown compared to those without CPDI distress.

## 4. Discussion

The aim of the present study was to investigate the psychological consequences of the COVID-19 pandemic during the lockdown period in patients with autoimmune arthritis and in controls.

At the moment, there are no specific antiviral treatments recommended for SARS-CoV-2, nor vaccines, thus, to reduce the spread of SARS-CoV-2, hard confinement measures, generally demonstrating to be effective, have been adopted worldwide. In Italy, the duration of the hard lockdown was nearly 2 months, from March 10 to May 3, much longer than the 10 days indicated in one study, as the time limit between tolerated and not-tolerated quarantine period [46]. The sudden limitation of social life, educational opportunities, isolation, and the inability to work, led to relevant psychological, social, health, and economic consequences; thus, the urgent need for mental healthcare has been proposed for the general population as a consequence of the lockdown [47].

We assumed that patients affected by chronic autoimmune arthritis could represent a vulnerable population at higher risk of peritraumatic stress as a consequence of the lockdown and the COVID-19 pandemic, due to a number of reasons. The first was the limitation of access to primary care or outpatient clinics, then, the fear of shortage of supplies of some immunosuppressive drugs used in COVID-19 positive patients, such as hydroxychloroquine and tocilizumab. It is possible that there has been a kind of disorientation in people, patients in particular, due to the dissemination of contradictory information often promoted by the media from flawed studies published without being peer-reviewed [48]. Furthermore, arthritis patients, knowing they are immune-depressed, may have experienced great fear of being more exposed to the SARS-CoV-2 infection; this issue has not yet been supported by evidence-based data, but this new type of apprehension may be added to the other, often unjustified fears, already described in over 17% of these patients, and may further fuel psychological distress [49]. The interpretation that the main psychological consequences of COVID-19-induced lockdowns in arthritis patients may principally be ascribed to the representation that patients have of their-own illnesses, more than the objectives and demonstrated arthritis-associated risks of infection, and severity of COVID-19; it may even be supported by the lack of influence of comorbidities on the perception of general health worsening, thus, underlying that the main driver of peritraumatic stress disorder is the worry by which the patients feel the disease more than the disease itself.

The psychological distress observed in autoimmune arthritis seems to be shared with some chronic diseases, including cardiovascular diseases [50], asthma and COPD [51], cancer [52,53], and psychiatric disorders [44,54,55]; whereas patients with other chronic conditions, such as multiple sclerosis [56] and, surprisingly, patients with diabetes, do not seem to have particularly suffered from the negative psychological consequences of COVID-19-induced lockdown, even though, for diabetes patients, their levels of adherence to treatment and lifestyle habits were reported to be significantly reduced [57]. The reason for these discrepancies among chronic conditions is difficult to interpret and may be ascribed to differences in study populations, such as age (i.e., older in arthritis than in multiple sclerosis or type I diabetes), gender (i.e., predominance of the female gender in arthritis than in type I diabetes), but also on the illness itself, such as severe inflammatory joint pain, stiffness and, consequently, functional limitation pathognomonic of autoimmune arthritis, which are closely dependent on tight medical monitoring, and can quickly impact the psychological well-being of these patients.

To the best of our knowledge, only one study addressed the psychological effects of the COVID-19 pandemic on 80 patients affected by inflammatory arthritis (mainly axial SpA) [58]. The authors found that 55% of patients presented mild to severe psychological distress, by using the Patient Health Questionnaire (PHQ-4) [59], with a higher prevalence among females. Moreover, females reported higher levels of decline in GH, mood disturbance, and increased disease activity. Despite the different questionnaires used in the two studies, PHQ-4 in the first and CPDI in the current one, the different percentages of patients with mild to severe psychological distress observed in the two works (55% in the first vs. 41% in the current one) are not statistically significant, as well as the percentages of patients presenting a worsening in GH and disease activity (25% vs. 36% and 29% vs. 37%, respectively). Having found similar results by different methodological approaches represents a further strengthening of the reliability of our results.

Our observation that among controls, 64%, 75%, and 68% did not present depression, anxiety, and stress, respectively, is very similar to the corresponding values of 67%, 81%, and 73%, reported by Mazza et al., with the same methodological approach, from a larger sample of the Italian general population [19]. This is reassuring, even though the sample size in this analysis is smaller. However, regarding the sociodemographic characteristics associated with depression, anxiety, and stress, we could not confirm that the female gender was a marker of vulnerability for all three expressions of psychological distress.

Our original hypothesis that arthritis patients may be more affected by the COVID-19 induced lockdown, was supported by the significantly higher CPDI mean score (25.6 vs. 19.3) and percentage (almost double) of patients with mild–moderate–severe CPDI (41% vs. 23%) in comparison to controls. Moreover, 36% of patients vs. 7% of controls reported a worsening in perceived GH during the lockdown, indicating that the higher peritraumatic distress suffered by the patients have strongly impacted their general health. These results are in line with a survey published in the 15 October 2020, issue of a major Italian daily newspaper, conducted by an Italian patient association (Apmarr), in which patients affected by chronic rheumatologic conditions reported increased levels of stress, depression, and anxiety during the lockdown, with 44% of them also reporting a worsening in perceived general health [60]. This description is all the more relevant as an expression of the patients’ perspective.

Furthermore, the results of multivariate analyses confirmed this observation, by demonstrating that being an “arthritis patient” is associated with higher CPDI score independently of depression, anxiety, and stress symptoms, or sociodemographic and lifestyle characteristics.

Of note, arthritis patients also had a higher CPDI score (25.6) in comparison to the Italian (22.3) and Chinese general populations (23.7) [17,45], and the prevalence of mild–moderate–severe CPDI among arthritis patients (41%) was higher than that reported for the Italian (30%) and Chinese general populations (35%) [17,45].

These results show that, in arthritis patients, mental health issues were significantly exacerbated by the stressful COVID-19 situation, and suggest that psychological support to these patients in a pandemic should be considered. The attempt to prevent, at least, the more severe forms of psychological distress envisages the need to release more clear and targeted messages that could empower these patients on the rationale of the imposed limits, increase their compliance, and avoid over-estimating their own risks of infection. In this delicate, unprecedented pandemic period, the role of research in scientific societies is crucial, as demonstrated by the recent EULAR statements [39]. Furthermore, in this scenario, telemedicine and tele-assistance can become potent tools that should be extensively implemented and adopted [61]. During the lockdown period, indeed, hospitals could receive only patients who needed urgent attention, nevertheless, patients with autoimmune arthritis need routine care and checkups to keep their diseases well-managed. Telemedicine can minimize patient exposure to infection risk, and keep the disease controlled and the patient reassured; thus, potentially decreasing anxious and depressive symptoms.

This study has some limitations and strengths. Data were all self-reported due to the lockdown measures and the administration of the questionnaire was via electronic support (to which not everyone was familiar, especially older people). Controls were not randomly selected from the population, but since our convenience sample was from relatives of people working in the same hospital, we ensured that the catchment area of cases and controls was similar. We considered the possibility of selecting a control group from spouses or caregivers of cases, but discarded it to avoid a selection bias due to the “burn-out” effect [62], since the person who lives with a family member suffering from arthritis may show anxiety for his/her relative. In order to adjust for the differences observed in the socio-demographic and lifestyle characteristics between cases and controls, we included them among the explanatory variables in the regression analyses.

Finally, the cross-sectional design of this study cannot establish a cause–effect relationship between lockdown measures and the development of psychological consequences. A possible approach to indirectly infer lockdown responsibility might be to compare the above reported rates in different populations of autoimmune arthritis patients, evaluated with the same methodological approach, the DASS21, before the COVID-19 pandemic. Despite studies being relatively scarce, Covic and coauthors, for RA patients, reported rates of severe/extremely severe depression, anxiety, and stress of 8.3%, 7.8%, and 9.8% [27], thus far below 19.2%, 23.1%, and 15.4% observed in RA patients in the current study (*p* < 0.05, *p* < 0.005, and NS, respectively).

The strength of this study is having described—through a specific methodological approach and comparison with convenience controls—that being an “arthritis patient” is significantly associated with higher COVID-19 peritraumatic distress, independently of depression, anxiety, and stress symptoms.

## 5. Conclusions

The results of this study demonstrate that, during the COVID-19 induced lockdown, arthritis patients presented a higher level of depression, anxiety, and stress symptoms, and also higher COVID-19 peritraumatic distress, than controls, which may have a negative influence on arthritis outcome.

Strategic, targeted intervention should be designed to reduce mental health problems and disease worsening in this vulnerable population. Future studies can further examine the value of positive personality traits as protective factors against acute stress [63].

## Figures and Tables

**Table 1:** Demographic, lifestyle, and clinical characteristics of controls and arthritis patients.

		%Controls (*n* = 100)	%Patients (*n* = 100)	*p* ^
Gender	Females	59.0	72.0	0.005
Age	<50 years	40.0	21.0	0.003
	≥50 years	60.0	79.0	
	*Mean (sd)*	*50.5 (20.4)*	*57.7 (12.5)*	0.003
BMI §	≥25	36.0	51.0	0.032
Education	Graduate	36.0	27.0	ns
Employment	Retired	30.0	26.0	ns
Smoking	Current smoker	10.0	25.0	0.005
Alcohol Use	Yes vs. no	75.0	59.0	0.016
Living	Alone	39.0	37.0	ns
Comorbidity	≥1	33.0	52.0	0.007
**Depression, Anxiety and Stress Scale-21 (DASS-21)**				
DASS-21 Depression	No (score 0–9)	64.0	58.0	0.029
	Mild-moderate (score 10–20)	32.0	27.0	
	Severe-extremely severe (score ≥ 21)	4.0	15.0	
	*Mean (sd)*	*7.7 (6.6)*	*10.3 (9.3)*	0.022
DASS-21 Anxiety	No (score 0–7)	75.0	62.0	ns
	Mild-moderate (score 8–14)	17.0	22.0	
	Severe-extremely severe (score ≥ 15)	8.0	16.0	
	*Mean (sd)*	*5.1 (5.8)*	*7.3 (7.7)*	0.020
DASS-21 Stress	No (score 0–14)	68.0	54.0	0.032
	Mild-moderate (score 15–25)	27.0	31.0	
	Severe-extremely severe (score ≥ 26)	5.0	15.0	
	*Mean (sd)*	*11.3 (8.2)*	*14.8 (8.8)*	0.005
**COVID-19 Peritraumatic Distress Index (CPDI)**				
	No (score 0–27)	77.0	59.0	0.022
	Mild-moderate (score 28–51)	22.0	38.0	
	Severe-extremely severe (score 52–100)	1.0	3.0	
	*Mean (sd)*	*19.3 (12.3)*	*25.6 (12.7)*	<0.001
Perceived General Health *	Worse	7.0	36.0	<0.001
	*Mean (sd)*	2.9 (0.5)	3.3 (0.8)	<0.001
Perceived Fatigue *	Worse	19.0	31.0	ns
	*Mean (sd)*	2.9 (0.8)	3.2 (0.8)	0.041
Perceived Emotional Status *	Worse	54.0	56.0	ns
	*Mean (sd)*	3.6 (0.8)	3.6 (0.8)	ns
Perceived Disease Activity *	Worse		37.0	
	*Mean (sd)*	/	3.3 (0.8)	
Perceived Joint Pain *	Worse		38.0	
	*Mean (sd)*	/	3.3 (0.9)	

§ BMI (Body Mass Index). * Change from the beginning of the lockdown. ^ According to type of variable, *p*-values calculated from χ^2^ or t-tests.

**Table 2:** Multivariate linear regression analysis. Adjusted effects of factors influencing coronavirus disease 2019 (COVID-19) Peritraumatic Distress Index (CPDI).

		b	95% CI		*p*
Cases		3.6	0.76	6.76	0.014
Gender	Females	1.53	−1.69	4.76	ns
Age	≥50	−1.58	−5.12	1.97	ns
BMI §	≥25	2.28	−0.90	5.35	ns
Education	Graduate	0.80	−2.38	3.99	ns
Employment	Retired	1.40	−2.23	5.02	ns
Smoking	Current smokers	0.97	−2.80	4.74	ns
Alcohol	Yes vs no	0.88	−2.25	4.01	ns
Living	Alone	−0.05	−2.89	3.00	ns
DASS-21 Depression	Mild–Moderate and Severe-extremely severe	4.70	0.81	8.59	0.018
DASS-21 Anxiety	Mild–Moderate and Severe-extremely severe	8.05	4.54	11.56	<0.001
DASS-21 Stress	Mild–Moderate and Severe-extremely severe	7.64	4.02	11.27	<0.001
Comorbidity	≥1	0.44	−2.67	3.56	ns

§ BMI (Body Mass index). DASS-21 (Depression, Anxiety and Stress Scale-21): Depression score≥ 10; Anxiety score ≥ 8; Stress score ≥ 15.

**Table 3:** Adjusted effects of factors associated with self-reported worsening of general health.

		OR	95% CI		*p*
Cases		9.01	3.11	26.07	<0.001
Gender	Females	2.98	0.98	9.02	ns
Age	≥50	0.39	0.13	1.17	ns
BMI §	≥25	0.68	0.26	1.76	ns
Education	Graduate	0.93	0.35	2.52	ns
Employment	Retired	1.34	0.43	4.13	ns
Smoking	Current smokers	0.80	0.29	2.26	ns
Alcohol	Yes vs. no	0.79	0.31	2.00	ns
Living	Alone	0.49	0.19	1.25	ns
Depression	Mild–Moderate and Severe-extremely severe	0.68	0.20	2.26	ns
Anxiety	Mild–Moderate and Severe-extremely severe	1.66	0.57	4.85	ns
Stress	Mild–Moderate and Severe-extremely severe	1.94	0.62	6.06	ns
Comorbidity	≥1	0.78	0.28	2.17	ns
CPDI ^	Mild-Moderate and Severe	5.56	2.06	14.99	<0.001

§ BMI (Body Mass Index). DASS-21 (Depression, Anxiety and Stress Scale-21): Depression score ≥ 10; Anxiety score ≥ 8; Stress score ≥ 15. ^ CPDI: COVID-19 Peritraumatic Distress Index: score ≥ 28.
